# Role of Arbovirus Infection in Arthritogenic Pain Manifestation—A Systematic Review

**DOI:** 10.3390/tropicalmed7110390

**Published:** 2022-11-21

**Authors:** Rafaella de Carvalho Cardoso, Bismarck Rezende, Allan Kardec Nogueira Alencar, Fabrícia Lima Fontes-Dantas, Guilherme Carneiro Montes

**Affiliations:** 1 Healthy Sciences School, Brazilian Institute of Medicine and Rehabilitation (IBMR), Rio de Janeiro 22631-002, Brazil; 2 Department of Pharmacology and Psychobiology, Roberto Alcântara Gomes Institute Biology (IBRAG), Rio de Janeiro State University (UERJ), Rio de Janeiro 20551-030, Brazil; 3 Department of Biomedical Engineering, Tulane University, New Orleans, LA 70118, USA

**Keywords:** arthritis, pain, arbovirus, disease, arthritogenic, arthralgia

## Abstract

The number of publications on the development of arthritic pain after CHIKV infection is increasing; however, there is still a gap in the pathophysiological mechanisms that explain these outcomes. In this review, we conducted a descriptive analysis of the findings of patients to understand their prognosis and to explore therapeutic options. Here, we searched the Cochrane, BVS, PubMed, and Scielo databases using the keywords “arthritis”, “pain”, “arbovirus”, “disease”, “arthritogenic”, and “arthralgia” during the 2000 to 2022 period. Descriptive analyses were conducted to understand the association between CHIKV infection and arthritogenic pain. The present study shows the persistence of acute phase signals for months, making the chronic phase still marked by the presence of arthralgia, often disabling under stimuli, such as temperature variation. CHIKV infection appears to be remarkably similar to rheumatoid arthritis, since both diseases share common symptoms. Once diagnosed, patients are mostly treated with analgesics, nonsteroidal anti-inflammatory drugs (NSAIDs), corticosteroids, and disease modifying anti-rheumatic drugs (DMARD). As there are no prophylactic measures or specific treatments for arboviruses, this study gathered information on the development and manifestations of arthritogenic pain.

## 1. Introduction

Viral infections with unpredictable clinical outcomes occur frequently worldwide. To date, 87 countries have reported autochthonous transmission of arbovirus [1]. An example is the epidemics of arboviruses Zika (ZIKV) and Chikungunya (CHIKV) that occurred in Brazil in recent years, becoming endemic and causing irreparable damage to the population [2,3]. While more than 80% of cases of ZIKV infection are asymptomatic or have very mild symptoms, this relationship is practically reversed in relation to CHIKV infection, which is usually symptomatic [4,5,6,7]. In addition to the clinical manifestations that are central nervous system (CNS) and peripheral nervous system (PNS)-specific [8,9], nonspecific neurological and/or rheumatological symptoms, such as myalgia, hypogeusia, articular pain, and general malaise, have been reported in patients after CHIKV infection [3,10,11]. Interestingly, it has been observed that the main symptoms of CHIKV infection do not differ between sexes. Nevertheless, the symptomatologic profile might vary from case to case depending on the preexistence of comorbidities, such as diabetes mellitus and osteoarthritis, and advanced age [12,13,14]. Persistent joint pain is a common manifestation of arthropod-borne viral infections and can cause long-term disability, although the precise mechanisms of Chikungunya disease progression from acute fever to the chronic phase and its correlation with arthralgia remain poorly understood [8,15].

The immune process linked to articular pain is triggered, at least in part, by post-infectious inflammation [16]. Notably, acute symptomatic CHIKV disease resembles other common known arbovirus-induced diseases, such as dengue virus (DENV) and ZIKV disease, independent of strain [17]. In this context, physicians tend to identify similarities in the clinical onset of rheumatoid arthritis after infection by CHIKV, Dengue fever virus, Yellow Fever virus, and Zika virus, and it is believed that the mechanisms involved in the chronicity of both diseases are similar [18,19]. Indeed, the inflammation induced by the presence of the virus in the joints has been implicated as a key factor for the development of acute and chronic polyarthritis following alphavirus infection [15]; however, the severity of this manifestation is based not only on viral tissue tropism but also on a possible autoimmune response [20]. Vijayalakshmi et al., 2017, showed that molecular mimicry between viral protein E1 and host proteins contributes to the development of arthritic manifestations by CHIKV through increased immune and inflammatory responses [21]. Another important study identified conserved regions of the alphavirus structural polyprotein that are homologous to human proteins involved in rheumatoid arthritis, which can be recognized by B cells and the MHC class II receptor [22].

Furthermore, some studies have suggested a neuropathic component of arthritogenic pain [8, 23, 24, 25, 26]. Other lines of evidence concern the involvement of nociceptive and neuropathic mechanisms in arbovirus infection [27,28]. Neuropathic pain is usually caused by injuries that damage somatosensory pathways from peripheral nerves to central structures, including the spinal cord and brain [29]. Importantly, CHIKV infection might promote demyelination, the most typical injury that harms the entire structure of a peripheral nerve [26]. Given that there are no effective prevention methods or treatments for arboviral diseases, the aim of the present study was to conduct a systematic review of the literature to advance the understanding of the pathophysiological mechanisms of arboviruses-induced joint pain and to identify methods for diagnosis and treatment.

## 2. Materials and Methods

This systematic review was registered in the International Prospective Register of Systematic Reviews (PROSPERO) under the number protocol: CRD42022367576, https://www.crd.york.ac.uk/prospero/ (accessed on 1 November 2022). Additionally, this work was written in accordance with the Preferred Reporting Items for Systematic Reviews and Meta-Analyses (PRISMA) guidelines.

### 2.1. Search Strategy

A literature search was conducted using five databases to identify studies that examined the association between arbovirus infections and arthritic pain. The main terms and expressions used in this research were arthritis, pain, and arbovirus. The following databases were used to perform a search: PubMed, LILACS, SciELO, Cochrane (search tools strategies MeSH, PICO, and advanced search), and BVS, published between January 2000 and December 2020. For the systematization of the research question, the PICO strategy (PICO—patient, intervention, comparison, and outcomes) was used, where P (patient) was positive for arbovirus infections and arthritogenic pain; I (intervention) was drugs used in the treatment; C (comparison) was between patients who developed chronic pain and those who did not have persistent pain, and O (outcome) was relevant findings that could justify the appearance of signs and symptoms and the effectiveness of the methodology used for treatment. The GRADE approach was applied to assess the quality of evidence for the set of available evidence and important findings that corresponded to the research question. The data found and described were considered satisfactory and met the criteria for the GRADE approach. Two autonomous persons ran the survey in duplicate.

### 2.2. Inclusion and Exclusion Criteria

The object of interest this study was to collect research results that included studies of cohort, case–control, and clinical cases in human experimental models. Only files published in English were chosen for reading. Studies published in conferences, systematic and narrative reviews, or editorials were excluded. Duplicate quests were excluded from the study.

In addition, a considerable number of the selected studies presented as a primary patient outcome the manifestation of arthritogenic pain with increased levels of important pro-inflammatory molecules such interleukins (IL-6, IL-8, and IL-13), tumor necrose factor alpha (TNF-α), monocyte chemoattractant protein-1 (MCP-1), and macrophage inflammatory protein 1 (MIP-1) in the analysis of synovial fluid from pain-affected joints. It has been further observed that pain was not present in all patients. However, in cases of pain manifestation, it persisted for days or even years. The different diagnostic methodologies (clinical and symptomatic approaches, direct analyses such as the PCR technique, or indirect analyses such as serological tests for IgG and IgM detection), as well as different therapeutic interventions (anti-inflammatory and analgesic drugs), might be classified as secondary events and were not considered the focus of the current work.

### 2.3. Study Selection and Data Extraction

Two authors proceeded with the study selection and extracted the data independently by using the same predetermined data extraction patterns. After deleting duplicates and articles in languages other than English, two phases were completed. The first step was to appraise titles and abstracts for the selection of pertinent scientific articles that met the eligibility criteria. The second step consisted of a full-text reading of the articles approved in the first step to conduct a more complete judgment in compliance with the inclusion and exclusion survey criteria. A consensus was achieved in cases of disagreement between the authors. A third evaluator was not required. The following data were extracted and recorded: Table 1—author, year, country, ethnicity, study design, diagnosis, treatments, and relevant key findings. Quality control of eligible studies was assessed using the Critical Appraisal Skills Program tools [30] for cohort and case–control studies by two independent authors (F.L.F.D or G.M.).

## 3. Results

### Study Selection

**Figure 1:**
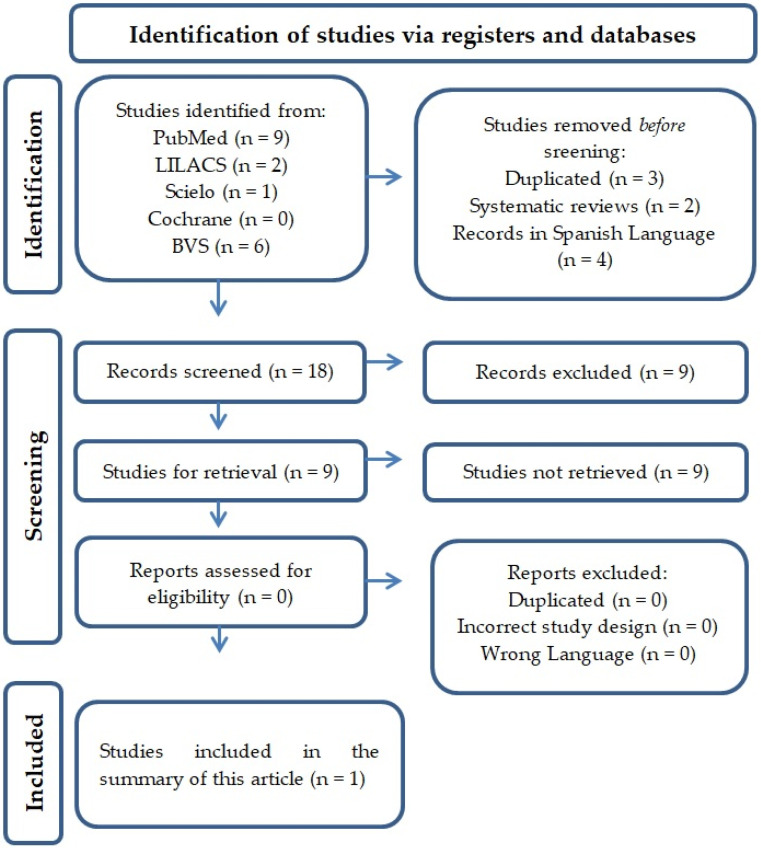
Illustrative flowchart of the rummage and article selection.

Eighteen articles were selected according to the search strategy. After applying the eligibility criteria, three articles were identified as duplicates, two were identified as systematic reviews, and four articles were written using languages other than English and were thus excluded of the final pool of studies (Figure 1). In total and based on data availability, we screened 18 studies by the title and abstracts and included 10 articles for a review of the observational analyses. There were 1036 patients in the total articles included, and most were female. The mean age of the patients was 50 years (6–87). Serum anti-CHIKV IgM and anti-CHIKV IgG levels were reported in 151 patients and were positive in only 19 and 28 patients, respectively, according to Pouriayevali et al., 2019 [23].

According to our search criteria, most records reporting CHIKV with joint signs/symptoms were from the European region (n = 387), followed by the Americas (n = 351) and the Asian continent (n = 289). Information about the symptomatologic profile of patients was documented in all studies, and the most frequently found symptoms were fever, rash, headache, myalgia, joint swelling, and pain due to a local inflammatory reaction.

Arthralgia and joint/articular pain were found in 98% (297 cases) of patients, according to Bouquillard et al., 2018. The most affected joints were the shoulders (168/300), elbows (37/300), wrists (216/300), hips (3/300), (161/300), ankles (212/300), and tarsus (152/300), according to the same author [24]. Patients diagnosed with rheumatoid arthritis were excluded from the study due to the similarity of symptoms with chronic pain after arbovirus infection. The duration of arthritis ranged from 2 days to 6 months among the nine cases who reported the time. The median treatment duration was approximately 10 days.

Different drug combinations were used as therapeutic strategies for mitigating signs and symptoms of infection. Corticosteroids in the management of inflammatory arthritis were effective at moderate doses and were used in the acute and chronic phases. In both the acute and chronic phases, joint pain was well-controlled with the administration of analgesics and NSAIDs. For patients unresponsive to this regular treatment, chloroquine proved to be a valid option, as evidenced by Bouquillard et al., 2018, and Ravindran et al., 2016 [24,32]. Ravindran et al., 2016, also demonstrated the efficacy of a triple combination of methotrexate, sulfasalazine, and hydroxychloroquine, which was superior to that of hydroxychloroquine monotherapy. According to the author, this drug combination led to a considerable reduction in pain levels by suppressing the expression of some pro-inflammatory cytokines, such as IL-6, IL-8, IL-13, TNF, MCP-1, and MIP-1, and for this reason, he considered triple therapy superior to monotherapy [32].

Bedoui et al., 2021, evaluated the expression levels of cPLA2α and mPGES-1 after therapy with methotrexate or dexamethasone. Methotrexate was not efficient in controlling the expression and activity of these enzymatic molecules; however, dexamethasone was shown to be able to normalize their levels [18].

An alternative method to drug treatment was pointed out by Ribeiro’s study, which used anti-inflammatory drugs and analgesics in parallel with the continuous local application of infrared laser, ultrasound, and TENS-burst. This article argues that the association between focal treatments using stimuli of different wave frequencies accelerates the healing process by collaborating in different ways in cell recovery, speed of nerve conduction, and collagen production and extensibility, which may reduce inflammation and, consequently, pain and arthritic stiffness [25].

## 4. Discussion

Although global concern has focused on the COVID-19 pandemic since 2020, arthropod-borne viruses continue to cause outbreaks. It is already known that arboviruses induce self-limiting symptoms in adults [3,32,36], and the occurrence of short- and long-term pain syndromes has become increasingly common in patients infected by the CHIKV [3,32,36]. The most common viruses causing arthritis and/or arthralgias are parvoviruses; hepatitis B virus; hepatitis C virus; Epstein–Barr virus (EBV); and tropical viruses, such as Dengue, Zika, and CHIKV [37,38].

Arthritis is defined as swelling and tenderness of one or more joints and involves inflammation, whereas arthralgia is a joint pain with no inflammatory cause [39,40]. Although both may share many symptoms, each condition also has distinctive characteristics that make them different [41]. CHIKV-induced arthritis involves joints and a common pattern of leukocyte infiltration (innate and adaptive immune response cells, such as monocytes and T and B cells), cytokine production, and complement activation and is closely dependent on the possible virus persistence on hidden sides [42,43].

In joints, macrophages, synovial cells, and chondrocytes produce the eicosanoid named prostaglandin E2 (PGE2), which is an important pro-inflammatory molecule involved in bone erosion and pain [44]. Additional cytokines produced in this context are IL-1β and TNF-α, biomolecules that stimulate the synthesis of PGE2 and enhance its local concentration [8,16]. Since this inflammatory milieu is observed in the joints, NSAIDs are usually chosen as a therapeutic option. The general mechanism of action of NSAIDs is the inhibition of cyclooxygenases 1 and 2, enzymes involved in the synthesis of prostaglandins. NSAIDs are commonly used to treat inflammatory diseases, such as rheumatic disorders, and aid in relieving pain and fever [45,46].

Serological tests, and clinical signs and symptoms have proven to be the basic and regular methods for diagnosing arboviruses. Furthermore, Zika and Chikungunya are infections with a very similar manifestation, and the clinical diagnosis may not be sufficient to differentiate them, requiring testing by other methods [4].

The most commonly used tests for differential diagnosis in the identification of serum levels of anti-antibodies (anti-IgM and anti-IgG) are ELISA (Mac-ELISA) and RT-PCR [47]. In addition, by performing immunohistochemistry, Bedoui et al., 2018, labeled a wide variety of primary antibodies used in cell cultures of synovial biopsy (hygroma), which originated from one of the patients in the study who had been in the chronic phase of the disease for at least 18 months, with persistent pain and relapsing arthralgia in more than one small joint. Moreover, RT-PCR from total RNA extracted from the same cell culture was performed. To verify the possible cytotoxic effects of the treatment, lactate dehydrogenase release was measured in the supernatants of HSF cell cultures 24 h post-treatment [18].

In addition, Blettery et al., 2019, combined serological tests and RT-PCR with joint imaging data acquired by Doppler ultrasonography. The Doppler ultrasound of painful joints revealed effusions in 92.8% of the examined joints (hands and wrists, ankles, and knees were involved, but shoulders and elbows were rarely affected). The majority (75.5%) of effusions were unilateral. No erosion was observed. Subcutaneous inflammatory infiltration (cellulitis) was observed in only a minor proportion of the participants [22]. In summary, RT-PCR was used in all studies that mentioned a diagnostic method, even in association with other alternative methodologies. This was the molecular technique of choice for the direct detection of viral RNA, which showed greater specificity and assertiveness. The Zika and Chikungunya epidemic in 2014–2016 in Brazil boosted the repositioning of drugs for the treatment of only acute symptoms; however, the identification of strategies for the management of persistent pain is still incipient [48]. The treatment is usually performed with common analgesics (e.g., acetaminophen) [49], NSAIDs (e.g., aspirin and ibuprofen) [50], dexamethasone [18], hydroxychloroquine, and DMARDs [32] such as methotrexate. Methotrexate acts by multiple mechanisms [51,52]. It decreases the nitric oxide production, stimulates adenosine release, activates the adenosine receptor A_2A_ (a physiological receptor with anti-inflammatory properties [53]), inhibits purine and pyrimidine synthesis, participates in transmethylation reactions, promotes the translocation of the nuclear factor-κB to the nucleus, and reduces the signaling pathway of the Janus kinase signal transducer and the transcription factor STAT [52,54,55]. Furthermore, other therapies, such as local applications of continuous ultrasound, infrared laser, and TENS-burst alone or in combination might be used as alternative or additional treatments [56,57]. Nevertheless, these techniques are only partially effective. Finally, according to the studies reviewed in this work, the risk of bias was considered low; most groups were heterogeneous; and patients were mostly female, despite having a variable age according to each study. Most cases were between 41 and 69 years old, and treatment efficiency measures were used in different ways, including morphological assessments; HAQ score; ultrasound; DAS28 ESR; X-ray; serological, immunological, and biochemical tests.

## 5. Conclusions

Conflicting explanations were found in the reviewed studies regarding the origin and causes of chronic arthritogenic pain in arbovirus infections. The serological and clinical examination results support that pro-inflammatory cytokine, TNF-α, and PGE2 may infiltrate the joints and generate symptoms similar to rheumatic arthritis. Furthermore, leukopenia, lymphopenia, neutropenia, and thrombocytopenia are evidenced.

Antivirals such as ribavirin are cited as possible drugs to control the viral load in patients infected in the acute phase of the infection. Moreover, the use of aspirin and other NSAIDs has been suggested as symptomatic treatment for the chronic phase. There are no findings that clearly demonstrate the circumstances for the chronicity of arbovirus-induced arthritogenic pain. Therefore, research in this area is desperately needed for a better understanding and elucidation of the issues addressed in this systematic review.

## Figures and Tables

**Table 1:** Characteristics of observational studies evaluating the association between arthritogenic pain and CHIKV infection.

Author; Year	Country	Ethnicity	Study Design	Diagnoses	Treatments	Relevant Key Findings
F. Rosso et al., 2018 [31]	Colombia	Colombian	Case Report and Literature Review	Not mentioned	Corticosteroids in the management of inflammatory arthritis	Patients in this study underwent organ transplantation. Some of them developed leucopenia, neutropenia, and thrombocytopenia, and all of them developed lymphopenia. None developed graft rejection or died in process.
E. Bouquillard et al., 2017 [24]	France	French	Original article	Serological test and clinical symptoms	After the acute phase, joint pain is well-controlled with analgesics and NSAIDs; corticotherapy may be effective at moderate doses; and Chloroquine salt are sometimes prescribed, particularly in the case of chronic joints that are non-responsive to analgesics and NSAIDs.	Chronic joint pain was associated with synovitis of the patients, affecting primarily the wrists, the proximal interphalangeal joints of the fingers, and the ankles. Attempts to detect the viral genome in joint fluid and synovial tissue using the RT-PCR technique were repeatedly unsuccessful.
Ravindran et al., 2016 [32]	India	Indian	Original article	Clinical symptoms and serological test	DMARD combination to Chikungunya arthritis (CA); triple combination with methotrexate, sulfasalazine, and hydroxychloroquine; monotherapy with hydroxychloroquine	Treatment with combination therapy leads to substantial improvement and reduces disability and pain in CA; levels of cytokines such as interleukin (IL)-6, IL-8 IL-13, and TNF; (MCP)-1; and (MIP)-1 also appear to play important roles in the pathogenesis of CA. Triple therapy is superior than monotherapy.
M. Blettery et al., 2019 [22]	France	French	Original article	Serological tests or PCR, then joints imaging studies by Doppler ultrasonography (DUS)	Not mentioned	DUS of painful joints revealed effusions in 92.8%of them (unilateral). Subcutaneous inflammatory infiltrations of the ankles were revealed at 29% of patients. Bone erosion was not observed.
A. Ribeiro et al., 2016 [25]	Brazil	Brazilian	Case Reports	Clinical symptoms.	Anti-inflammatory and analgesics drugs; in parallel, for local joints, applications include continuous ultrasound, infrared laser, and TENS-burst	The association of ultrasound, infrared laser, and TENS may accelerate the healing process by collaborating in different ways in cell recovery, speed of nerve conductions and collagen production, and extensibility. It could reduce inflammation, pain, and joints stiffness.
M.H. Pouriayevali, et al., 2019 [23]	Iran	Iranian	Original article	Serological test (ELISA and PCR tests) and clinical symptoms	Not mentioned	Correlation between abroad travel history and CHIKV infection; also, Iran-5300 strain showed a rare non-synonymous substitution T/C at nucleotide 10,560.
Y. Bedoui et al., 2021 [18]	France	French	Original article	Serological test, RT-PCR, immunohistochemistry, cytotoxicity assays	Methotrexate and dexamethasone	PIC and CHIKV enhanced mRNA expression of COX-2; PIC increased the mRNA levels of cPLA2α and of mPGES-1, two other central enzymes in PGE2 production; IFNβ upregulated cPLA2α and COX-2 transcription levels; MTX failed to control the expression of all these enzymes, but dexamethasone was able to control the capacity of pro-inflammatory cytokines.
M. Agrawal et al., 2019 [33]	India	Indian	Original article	Not applicable	Not applicable	The modulation of AKT3 induces the TNF-α-mediated autophagy and cytokine secretion. Additionally, AKT3 has been reported to act via PI3K/AKT/mTOR pathway, which activates the antiapoptotic genes and sensitizes the fibroblasts to TNF-α and TRAIL-mediated apoptosis. Therefore, during CHIKV infection, suppression of the robust inflammatory response may be regulated by the induction of hsa-miR-4717-3p through AKT3 gene target.
J. J. Hoarau et al., 2010 [34]	France	French	Original article	RT-PCR, Mac-ELISA, Clinical examinations and biological symptoms; PFU evaluation, immunochemistry and Western blot analysis	Methotrexate	CHIKV (mRNA and proteins) persisting in synovial macrophages could contribute to tissue injuries, apoptosis in vitro, fibrosis, and a polarized inflammatory response reminiscent of rheumatoid arthritis. The expression of immunoregulatory cytokines, such as IL-10 and TGF-β1, was demonstrated at T0 (time 0, considered before symptoms appearance) and M6 (Month 6).
M.d.R.Q. Lima et al., 2021 [35]	Brazil	Brazilian	Original article	Mac-ELISA, RT-PCR	Not mentioned	The Euroimmun anti-CHIKV IgM ELISA test showed 100% sensitivity and 25.3% specificity due to cross reactivities observed with dengue. IgM positive and acute cases of dengue, the assay showed cross-reactivity of 46.7% and 31.6%, respectively, and so, molecular tests, such as RT-PCR, was used as an option to confirm cross-reactivity or not.
